# 质谱成像技术在环境污染物分析及毒性研究中的应用进展

**DOI:** 10.3724/SP.J.1123.2023.11005

**Published:** 2024-02-08

**Authors:** Fang LI, Qian LUO

**Affiliations:** 中国科学院深圳先进技术研究院, 广东 深圳 518055; Shenzhen Institute of Advanced Technology, Chinese Academy of Sciences, Shenzhen 518055, China

**Keywords:** 质谱成像, 空间代谢组学, 环境污染物, 可视化分析, 空间分布, 毒性, 综述, mass spectrometry imaging (MSI), spatial metabolomics, environmental pollutants, visualization analysis, spatial distribution, toxicity, review

## Abstract

环境污染暴露与人类健康和疾病发生发展密切相关。污染物进入生物体内会被代谢和蓄积,通过与多种生物分子相互作用产生毒性效应。阐明污染物在生物体内的分布特征,明确其引发的毒性效应并发现关键毒性生物标志物,是环境毒理学研究的重要内容。外源性污染物在不同组织和器官中的代谢程度和累积情况存在差异,内源性生物分子合成和累积也具有精准的空间分布,因此对它们在生物体内的空间分布进行原位可视化分析具有重要意义。质谱因出色的定性和定量能力成为化合物分析的关键技术,以它为基础的质谱成像技术(MSI)是一种新兴分子影像技术,单次分析可提供组织切片上数百种内源性和外源性化合物的结构、含量和空间分布信息。该技术具有免标记、无需复杂样品前处理、高灵敏和高通量等优点,已被用于生物学、药学和临床研究中。近年来,MSI技术已成为环境分析和毒理学研究中最具前景的新技术之一,不仅可表征环境污染物在生物体整体和特定器官内的时空分布特征,还可通过开展空间代谢组学解析内源性代谢物对暴露的应答情况。本文介绍了常见MSI技术电离源的原理和特点、常用质量分析器及分析流程,对其在环境污染物分析和毒理学研究方面的应用进行了全面综述,并对未来发展方向进行了展望。

## Abstract (en)

Environmental exposures have significant impacts on human health and can contribute to the occurrence and development of diseases. Pollutants can enter the body through ingestion, inhalation, dermal absorption, or mother-to-child transmission, and can metabolize and/or accumulate in different tissues and organs. These pollutants can recognize and interact with various biomolecules, including DNA, RNA, proteins, and metabolites, disrupting biological processes and leading to adverse effects in living organisms. Thus, it is crucial to analysis the exogenous pollutants in the body, identify potential biomarkers and investigate their toxic effects. Numerous studies have shown that the metabolism rate of environmental pollutants greatly differs in various tissues and organs, their accumulation is also heterogeneous and dynamically changing. Moreover, the synthesis and accumulation of endogenous metabolites exhibit precise spatial distributions in tissues and cells. Mapping the spatial distributions of both pollutants and endogenous metabolites can discover relevant exposure biomarkers and provide a better understanding of their toxic effects and molecular mechanisms. Mass spectrometry is currently the preferred method for the qualitative and quantitative analysis of various compounds, and has been extensively utilized in pollutant and metabolomics analyses. Mass spectrometry imaging (MSI) is an emerging technology for molecular imaging that combines the information obtained by mass spectrometry with the visualization of the two- and three-dimensional spatial distributions of various molecular species in thin sample sections. Unlike other molecular imaging techniques, MSI can perform the label-free and untargeted analysis of thousands of molecules, such as elements, metabolites, lipids, peptides, proteins, pollutants, and drugs, in a single experiment with high sensitivity and throughput. Different MSI technologies, such as matrix-assisted laser desorption ionization mass spectrometry imaging, secondary ion mass spectrometry imaging, desorption electrospray ionization mass spectrometry imaging, and laser ablation inductively coupled plasma mass spectrometry imaging, have been introduced for the mapping of compounds and elements in biological, medical, and clinical research. MSI technologies have recently been utilized to characterize the spatial distribution of pollutants in the whole body and specific tissues of organisms, assess the toxic effects of pollutants at the molecular level, and identify exposure biomarkers. Such developments have brought new perspectives to investigate the toxicity of environmental pollutants. In this review, we provide an overview of the principles, characteristics, mass analyzers, and workflows of different MSI techniques and introduce their latest application advances in the analysis of environmental pollutants and their toxic effects.

人类多数疾病的发生是由环境和基因因素共同驱动,超过70%的疾病与环境污染有关^[1]^。明确环境污染暴露与人体健康及特定疾病间的因果关系,锁定关键风险因子,有助于疾病的精准防控。重金属、颗粒物和有机污染物等的暴露从妊娠期开始贯穿整个生命周期,可通过呼吸、饮食、皮肤接触和母婴传递等途径进入体内,并对健康产生潜在威胁。污染物对健康的影响十分复杂,进入体内后由血液或其他体液转送到机体各个组织和器官,进行代谢和蓄积,通过与多种生物分子相互识别和交互作用而产生毒性效应^[2]^。阐明外源性污染物在生物体内和特定器官微区中的分布特征及暴露引发的毒性效应是环境毒理学研究的重要内容。除了常规毒理学手段,组学技术也是目前解析污染物毒性效应的有力工具。内源性代谢物不仅可以放大相关基因和蛋白质的变化,还可作为调节剂直接调控生理过程和表型,定量描述代谢物变化的代谢组学技术已被用于多种环境污染物的毒性效应、作用机制和生物标志物研究中^[3]^。

组织中外源性污染物的分布呈不均匀且动态变化,内源性代谢物合成和累积也具有精准的空间分布,这与组织的细胞异质性和结构复杂性有关^[4]^。常用的正电子发射断层扫描(PET)、磁共振成像(MRI)、荧光成像和拉曼成像等分子成像技术具有高灵敏度、高分辨率、实时性和非侵入性等优势,但难以在无标记的基础上同时实现组织中数百种及以上化合物的可视化分析。质谱技术是污染物和内源性代谢物定性和定量分析最主要的技术手段。常规分析时需将生物样品和组织匀浆处理以获得丰富的分子信息,但此操作会导致待测物的空间信息丢失。应运而生的激光显微切割技术通过对特定位置细胞和组织进行取样而保留待测物的空间位置信息,是目前空间多组学分析的关键技术,但后续生物分子的空间分布重构较为复杂。质谱成像技术(mass spectrometry imaging, MSI)是一种基于质谱分析的新型分子影像技术,能够直接扫描生物组织切片,从而获得大量已知或未知内源性和外源性化合物(如小分子代谢物、蛋白质、多肽、脂质和药物等)的化学结构、相对含量和空间分布信息^[5]^。Huang等^[6]^利用此技术对氯化石蜡和六溴环十二烷在斑马鱼器官中的毒代动力学和代谢毒性效应进行了原位表征。本文基于不同MSI技术原理和特点,系统综述了MSI技术在环境污染物分析和代谢毒性效应解析方面的应用进展。

## 1 质谱成像技术概述

### 1.1 MSI技术原理

MSI技术是一种以质谱为基础的表征离体组织和细胞中元素和化合物空间分布的免标记影像技术。通过一个聚焦的电离源(如离子枪、激光、分子束等)直接扫描样品使其表面分子解吸离子化,通过质量分析器检测各个像素点的质荷比(*m/z*)和离子强度,最终由成像软件结合质谱数据和位置信息对待测物的空间分布进行重构和可视化^[7]^。该技术可提供生物整体、组织微区、单细胞或亚细胞尺度待测物的定性、相对定量和定位信息。MSI技术于1997年首次用于大鼠垂体中激素肽和胰腺组织中胰岛素的可视化分析,随着电离源技术和质量分析器的发展与进步,目前已在基础医学、药学、环境科学等多个领域得到应用。

### 1.2 常用质谱成像技术类型

目前常用MSI技术根据离子化方式的不同可分为基质辅助激光解吸电离质谱成像技术(matrix assisted laser desorption ionization mass spectrometry imaging, MALDI-MSI)、解吸电喷雾电离质谱成像技术(desorption electrospray ionization mass spectrometry imaging, DESI-MSI)、二次离子质谱成像技术(secondary ion mass spectrometry imaging, SI-MSI)和激光剥蚀电感耦合等离子体质谱成像技术(laser ablation inductively coupled plasma mass spectrometry imaging, LA-ICP-MSI)等^[8]^。这些技术在适用分析对象和空间分辨率等方面各具特点,详见表1。

**表1:** 常见质谱成像技术的比较

MSI technology	Mass analyzer	Analytes	Vacuum condition	Spatial resolution/μm	Disadvantages
MALDI-MSI	TOF MS	metabolites, proteins, peptides,	vacuum	up to 5.0	matrix application, ionic suppression in low
		drugs and pollutants	ambient	up to 1.4	mass compound analysis
DESI-MSI	TOF MS	<2000 Da compounds	ambient	up to 10	low spatial resolution
SI-MSI	TOF MS	elements	vacuum	up to 0.05	instrument expensive, ionic fragmentation
		<1000 Da compounds		up to 1.0	
LA-ICP-MSI	TOF MS	elements	ambient	up to 5.0	matrix and fractionation effects

MALDI-MSI: matrix assisted laser desorption ionization mass spectrometry imaging; DESI-MSI: desorption electrospray ionization mass spectrometry imaging; SI-MSI: secondary ion mass spectrometry imaging; LA-ICP-MSI: laser ablation inductively coupled plasma mass spectrometry imaging; TOF MS: time-of-flight mass spectrometry.

#### 1.2.1 MALDI-MSI技术

MALDI-MSI是目前应用最广泛的MSI技术,通过激光束照射基质喷涂过的样品表面,基质吸收激光能量并传递给待测物使其解吸与离子化,适用于生物样本表面代谢物、多肽、蛋白质和药物等化合物的可视化分析^[9]^。除了激光激发波长和强度外,基质选择对于MALDI离子化效率至关重要。常用基质包括2,5-二羟基苯甲酸(DHB)、*α*-氰基-4-羟基肉桂酸和芥子酸等^[10]^,但它们存在背景峰干扰高、选择性弱和相对分子质量较低的化合物电离效率低等问题。为此,研究者们开发了9-氨基吖啶、*N*-(1-萘基)乙二胺二盐酸盐、1,5-二氨基萘、烷基化DHB和3-氨基邻苯二甲酰肼等有机小分子^[11,12]^和金、银、碳及其氧化物等无机纳米材料新型基质^[13,14]^。近期,我们团队开发了一类具有甲基吡啶鎓甲醛阳离子结构的反应性试剂,可与胆固醇的羟基进行亲核加成反应促进其解吸和电离^[15]^。该技术成像空间分辨率取决于基质结晶尺寸和激光光斑尺寸,真空MALDI分辨率为5~10 μm,大气压MALDI分辨率最高可达1.4 μm^[16]^。最新的激光诱导后电离(MALDI-2)技术将空间分辨率提升至0.6 μm,灵敏度也提高了1~3个数量级^[17]^。

#### 1.2.2 DESI-MSI技术

DESI-MSI是一种常压常温、敞开式MSI技术,高速雾化气带动溶剂在高电压下形成电喷雾,直接吹扫样品表面溅射出含待测物的次级带电液滴束,溶剂快速蒸发将电荷转移给待测物并使其形成气态离子,通过离子传输管进入质量分析器^[18]^。成像效果与电喷雾溶剂组成和流速有关,会影响样品表面待测物的溶解度、扩散性和离子化效率,主要用于分子质量2000 Da以下化合物的分析。成像空间分辨率受喷雾装置限制,喷嘴与样品表面和质谱进样口间的距离和角度对空间分辨率和信号强度影响极大。现有商业仪器空间分辨率为20 μm,对于大面积生物样品和非生物样品中化合物成像分析独具优势。Laskin等^[19]^采用2个二氧化硅毛细管组装的纳喷解吸电喷雾电离源(Nano-DESI),通过将探针与流动相形成流动溶剂桥直接接触样品表面进行成像分析,离子传输效率大大提升且易产生多电荷蛋白质离子,最新研究将此技术空间分辨率降至7 μm^[20]^。

#### 1.2.3 SI-MSI技术

SI-MSI技术可用于元素和分子质量小于1000 Da的疏水性化合物的可视化分析,是目前空间分辨率最高的MSI技术。在高真空环境下,利用聚焦的高能初级离子束轰击样品表面,产生次级离子进入质量分析器进行分析,已被用于地球科学、材料科学和生命科学等领域^[21]^。根据离子束类型和束流大小可分为动态SI-MSI和静态SI-MSI。前者采用单原子离子束(如CS^+^、O^-^和Ar^+^等)进行元素成像分析,纳米SI-MSI成像空间分辨率可达50 nm;后者以多原子或团簇原子离子束(如 $A{u_{3}}^{+}$ 、 $B{i_{3}}^{+}$ 、 ${C_{60}}^{+}$ 、 $A{r_{2500}}^{+}$ 和 $A{u_{400}}^{+}$ 等)进行化合物和部分元素成像分析,空间分辨率为μm尺度。SI-MSI技术分析分子质量较大化合物时存在二次离子产额较低且离子碎片化严重。Tian等^[22]^开发的可用于SI-MSI系统的70 keV (CO_2_ ${{)}_{n}}^{+}$ (*n*>10 000)气团离子束,化学破坏性较低,可检测化合物分子质量范围扩大到了3000 Da,空间分辨率降至1 μm。

#### 1.2.4 LA-ICP-MSI技术

与前三者技术不同,LA-ICP-MSI技术发展最早且最为成熟,主要用于元素可视化分析。通过将激光束聚焦于样品表面使微区样品熔蚀气化,由载气将样品微粒送至等离子体中进行原子化并电离,最后进入质量分析器进行检测,具有分析速度快、进样效率高、可进行多元素同时分析并提供同位素信息的优势。自2003年首次被用于表征羊肝脏组织中Cu的分布后,LA-ICP-MSI技术开始被广泛用于生命科学领域中的元素成像分析^[23]^。由于衍射极限和透镜数值孔径限制,LA-ICP-MS技术的空间分辨率在μm尺度。Meng等^[24]^研制的三通结构样品剥蚀池使LA-ICP-MS成像系统空间分辨率降低至0.4 μm,并实现了对小鼠小肠组织和Hela细胞内药物和纳米材料的可视化分析。近年来,将LA-ICP-MS技术与免疫组织化学技术相结合的质谱流式成像系统可在单细胞层面实现数十个蛋白质和其他生物分子的成像分析,扩大了可检测目标物范围。

#### 1.2.5 其他技术

随着离子化技术的革新,一系列解吸/后电离和等离子体新型电离技术不断出现。空气动力辅助离子源(AFAI-DESI)利用空气流实现大气压中离子或带电液滴的远距离传输,在质谱入口富集带电液滴,提高了离子化效率,并扩展了待测样品的应用空间^[25]^。激光消融电喷雾电离源通过先将待测物激光消融解吸后再进行电喷雾离子化,可进行大分子质谱化合物的成像分析^[26]^。介质阻挡放电电离源通过在两个放电电极之间放置绝缘介质,施加交流电压使两电极间的惰性气体或混合气体电离形成稳定低温等离子体,并对载体上的待测物进行解吸和离子化^[27]^。低温等离子体电离源则是利用气体(He、Ar或空气)在电场放电作用下产生的低温等离子体喷射样品表面使待测化合物解吸并离子化^[28]^。这些离子化技术在检测目标物类别、空间分辨率、电离效率、稳定性和灵敏度等方面得到了不同程度的提升和改进。

### 1.3 常见MSI质量分析器

质量分析器是成像质谱仪的核心组成部分,高质量分辨率和质量精度是进行化合物准确注释的必要条件。MSI技术多采用高分辨质量分析器。飞行时间质谱仪(TOF MS)因灵敏度高、分析速度快、可同时检测多个分子且理论上无质量检测上限,成为MSI技术最常用的质量分析器。静电场轨道阱质谱、傅里叶变换离子回旋共振质谱和多反射TOF MS的质量分辨率> 100000、质量精度< 10^-6^ (1 ppm),常与DESI、常压MALDI和AFAI-DESI等离子化技术相结合^[29⇓-31]^。近年来,离子淌度技术因对结构类似物和同分异构体的分离能力强在MSI系统中表现出独特优势,显著提高了化合物的鉴定准确性和成像精度,尤其在脂质组可视化分析方面。Djambazova等^[32]^将捕集离子淌度技术用于MALDI-MSI系统,发现峰容量提高了250%,实现了对脂质*sn*位置、酰基链和C=C位置异构体的分离。近日,研究者将淌度分离后的母离子不经质量隔离而完全碎裂并进行非依赖数据采集,结合智能谱图解卷积算法实现了多种脂质的结构解析和组织空间分布可视化,在不增加成像分析时间的情况下显著提升了脂质组结构解析能力^[33]^。

### 1.4 质谱成像技术分析流程

MSI分析工作流程为样品制备-质谱数据采集-数据处理与可视化分析,见图1。以小鼠肾脏组织成像为例,首先收集新鲜肾脏组织立即冷冻,冰冻切片(厚度5~20 μm),并进行切片预处理;以网格划分模式进行质谱分析获得各像素点质谱图,经数据预处理后进行鉴定、统计分析和可视化。

**图1:**
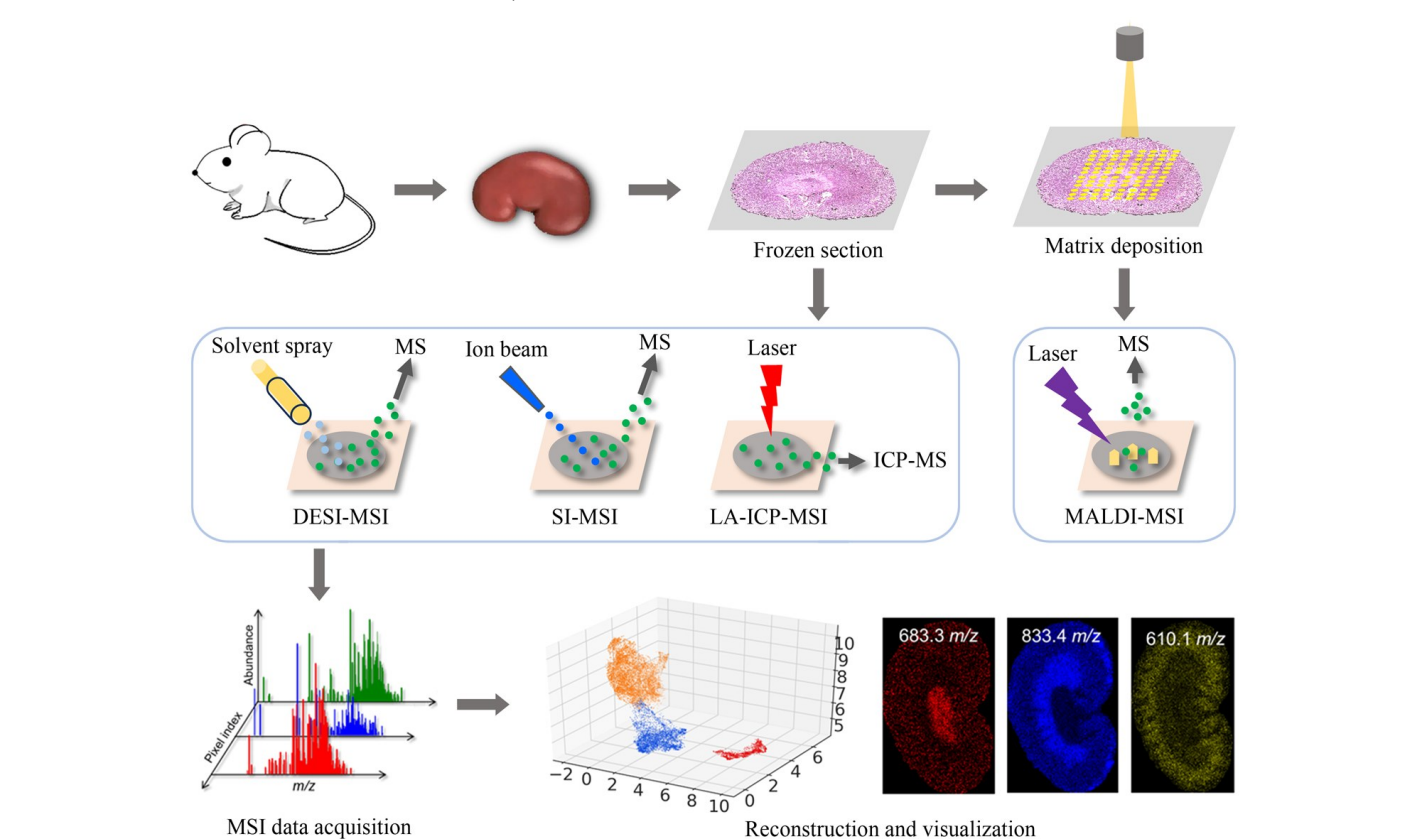
常见MSI技术及工作流程

维持组织形态不变和组织完整性对物质分子信号强度和定位十分重要,同时组织切片操作和保存不当也会导致表面分子降解移位影响质谱成像的准确性和真实性。数据采集质量取决于成像质谱仪的性能,根据需求选择离子化技术,高分辨率和高精度质量分析器是分子鉴定的必要条件。低极性、难电离物质分子是MSI可视化分析的难点,研究者们采用酯化、酰化、加成、取代、氧化等化学原位衍生化技术增强它们的电离效率,并改变分子质量与背景信号进行区分。Shariatgorji等^[34]^使用具有1-甲基-2-氟代吡啶阳离子结构的试剂对一级胺、二级胺和酚类等多种神经递质进行原位衍生化,实现了脑组织中神经递质的全景式可视化分析。海量成像数据经基线校正、峰提取、峰对齐与峰校正、峰归一化等数据预处理后进行化合物重构和可视化^[35]^,可用的成像软件包括SCiLS lab、HDI和MassImager^TM^等商业化软件和MALDIquant、Cardinal、rMSIproc8、MSireader和Datacube Explorer等开源软件。此外,以MSI数据为基础,与组织学染色或影像学数据多模态融合实现分子信息与解剖结构间的空间匹配,可更全面解读污染物的毒性效应^[36]^。

## 2 质谱成像技术在环境污染物分析研究中的进展

外源性的污染物进入生物体内后会分布到全身各个组织和器官,在不同组织和器官中的代谢速度和累积量不同。生物体内污染物分布特征描述和量化是环境毒理学研究的重要内容。MSI技术已被用于模式动物和植物中多种重金属、颗粒物和有机污染物的可视化分析,见表2。

**表2:** MSI技术在生物体内环境污染物分布特征研究中的应用

Classification	Pollutants	Samples	MSI technology	Spatial resolutions/μm	Ref.
Heavy	CH_3_Hg(Ⅱ), iAs(Ⅲ), Ag(Ⅰ) and Cd (Ⅱ)	zebrafish	LA-ICP-MSI	20	[37]
metals	Cd	worm strains	LA-ICP-MSI	8	[38]
	Cd	sunflower	LA-ICP-MSI	110	[39]
Particulates	Ag nanoparticle	mouse kidney	LA-ICP-MSI	20	[40]
	PbO nanoparticles	mouse	LA-ICP-MSI	20	[41]
	CeO_2_ nanoparticles	mouse	LA-ICP-MSI	100	[42]
	La_2_O_3_ nanoparticle	Pfaffia glomerata	LA-ICP-MSI	100	[43]
	graphene and graphene oxide particle	soybean plants	LA-ICP-MSI	110, 80	[44]
	black carbon	mouse	MALDI-MSI	20	[45]
Organic	perfluorooctanoic acid	zebrafish	MALDI-MSI	50	[46]
pollutants	perfluorooctane sulfonate	mouse	MALDI-MSI	50	[47]
	dinotefuran and acetamiprid	honeybees	MALDI-MSI	30	[48]
	imidacloprid, methiocarb	Cotoneaster horizontalis	DESI-MSI	100, 50	[49]
	dimethoate	Kalanchoe blossfeldiana			
	chlorinated paraffins and hexabromocyclododecane	zebrafish	AFAI-DESI-MSI	200	[6]

AFAI-DESI: air flow assisted-desorption electrospray ionization.

### 2.1 重金属

重金属是一类重要的环境污染物,进入体内后不易被排出。Zarco-Fernández等^[37]^采用LA-ICP-MSI技术研究了Cd(Ⅱ)、CH_3_Hg(Ⅱ)、Ag(Ⅰ)和iAs(Ⅲ)暴露48 h后在斑马鱼中的分布特征,发现Cd(Ⅱ)主要富集在眼睛处,CH_3_Hg(Ⅱ)则主要富集在消化道,其余两个金属离子均未进入体内。Wang等^[38]^发现TiO_2_纳米颗粒能显著增加线虫生殖腺和胚胎中Cd的负荷量,促进Cd通过种系转移到下一代。Pessôa等^[39]^采用此技术发现Cd可从土壤转移到葵花仁种子中,且子叶中含量最高。由于缺乏与待测样品基体匹配的标准物质、易受基体效应、分馏效应和质量歧视等影响,LA-ICP-MSI技术难以对组织内元素进行绝对定量分析,可采用油墨印刷、干燥液滴、明胶薄膜基质等策略进行校正。

### 2.2 颗粒物

日常生活环境中存在着大量的颗粒物,如PM2.5和人造纳米颗粒等。研究者们采用LA-ICP-MSI技术解析纳米颗粒在小鼠器官中的分布特征,发现Ag纳米颗粒主要聚集在小鼠肾脏肾皮质及其与髓质交界区域,且后者处的含量明显高于前者^[40]^, PbO纳米颗粒呼吸暴露后除了肺组织还会进入小鼠肝脏和肾脏组织中^[41]^, CeO_2_纳米颗粒主要累积在小鼠肝脏Kupffer细胞和小叶周围,在脾脏的累积与暴露时长有关,短期暴露主要累积在小鼠脾脏边缘区,长期暴露会深入白髓区^[42]^。此技术还被用于解析颗粒物在植物中的分布和迁移规律,研究发现La_2_O_3_纳米粒子主要分布在小球藻叶片的主静脉中^[43]^,石墨烯纳米颗粒物富集在大豆叶片中央和主静脉附近及根皮质层,而氧化石墨烯则表现为更加均匀地向叶片周围扩散和富集在根筛管部^[44]^。黑碳污染物可不使用基质直接进行MALDI-MSI分析,且质谱指纹图谱不受其来源和形态影响,研究发现PM2.5来源的黑碳颗粒主要累积在小鼠肺部,且短期内几乎不会向其他组织和器官中转移^[45]^。

### 2.3 有机污染物

有机污染物种类繁多,大多数具有生物累积性和持久性。Bian等^[46]^采用MALDI-MSI技术发现全氟辛酸能进入斑马鱼多个组织,在胆囊、肝脏、心脏、肾脏与肠道中的动态累积趋势一致,在鱼鳔、脊骨、鳃、肌肉与大脑一致,但与前者不同。Chen等^[47]^利用此技术发现全氟辛烷磺酸主要富集在小鼠肾脏肾盂和外皮质区,在髓质和内皮质区含量较少。将此技术用于蜜蜂体内烟碱类农药毒代动力学研究,发现呋虫胺(dinotefuran)和啶虫脒(acetamiprid)农药经口暴露2 h后能迅速穿透蜜蜂的各种生物屏障分布在全身部位,并在肠道进行富集,6 h后呋虫胺均匀分布在蜜蜂体内,而啶虫脒则已降解50%^[48]^。此外,MSI技术还被用来解析植物中农药的迁移规律。Gerbig等^[49]^利用DESI-MSI技术研究发现吡虫啉(imidacloprid)在平枝栒子(*Cotoneaster horizontalis*)叶片边缘积聚,而甲硫威(methiocarb)分布较均匀;土壤中的乐果(dimethoate)25天时主要分布在长寿花(*Kalanchoe blossfeldiana*)运输系统中,60天时则主要分布在叶片中。

总体来说,MSI技术可绘制外源性环境污染物在生物组织中的空间分布特征,为污染物的毒代动力学和植体内迁移规律研究提供了更为直接的证据。与重金属成像分析相比,生物组织中有机污染物的离子化效率低,目前商业化成像仪器仅可对少量有机污染物进行可视化分析。最新研究通过在AFAI-DESI喷雾溶剂中加入四苯基氯化膦提高多卤化烷基化合物的离子化效率^[6]^,发现短链氯化石蜡、中链氯化石蜡和六溴环十二烷可进入斑马鱼的多个组织中,前两者在鳃、肝脏和心脏中含量最高,后者在肾脏中也高度富集。

## 3 质谱成像技术在环境污染物毒性效应研究中的进展

### 3.1 MSI技术用于模式生物中内源性代谢物的空间分布特征研究

环境污染物毒性效应研究常选与人类基因高度相似的模式生物进行暴露实验,包括大小鼠和斑马鱼等,基于MSI的空间代谢组学技术可用于这些模式生物整体或特定组织中代谢物的可视化分析^[50]^。Pang等^[31]^采用AFAI-DESI-MSI技术表征了大鼠脑组织中的神经递质、嘌呤、有机酸、多胺、胆碱和碳水化合物的微区分布,如乙酰胆碱在大脑皮质中的丰度最高,*γ*-氨基丁酸在中脑、嗅球和下丘脑丰度较高,多巴胺主要分布在纹状体,而组胺则主要分布在海马和丘脑中,在此基础上绘制了脑组织中不同微区间的代谢网络图谱。脑组织中脂质也具有特异的微区分布特征^[51]^,我们团队采用DESI-MSI技术发现小鼠大脑中脂质分布特征主要分为三类:白质和丘脑区高丰度、皮层和海马区高丰度及脑室区高丰度。斑马鱼因体型较小,采用明胶和羧甲基纤维素(CMC)包埋后可对胚胎、幼鱼和成鱼整体及特定组织进行MSI分析。研究发现,部分磷脂酰胆碱(PC)和脂肪酸在斑马鱼眼睛、脑、鳃、肠中的含量明显高于其他组织,如PC(34∶1)在眼睛、脑和脊柱中丰度较高,PC(o-32∶0)在鳃中丰度较高,PC(30∶0)则在眼睛视网膜和晶状体中高度富集,脂肪酸则主要分布在鳃和肠道中^[52,53]^。Duenas等^[54]^采用MALDI-MSI技术也发现新受精斑马鱼胚胎中常见磷脂在胚盘内和卵黄边界均呈对称分布,PC特异性富集在卵黄和囊胚区,磷脂酰乙醇胺(PE)富集在胚盘中,磷脂酸(PA)和磷脂酸(PI)富集在囊胚区,磷脂酰丝氨酸(PS)则富集在胚胎壁上。此外,研究者们也采用MSI技术建立了特定脂质与大型溞、昆虫等模式生物解剖特征的关联性^[55]^。

### 3.2 MSI技术在环境污染物毒性效应研究中的应用

环境污染物暴露会引起生物体内生物学过程受损,对结构复杂且异质性的靶器官和效应器官的毒性效应和机制更为复杂。基于MSI技术的空间代谢组学已被用于环境污染物毒性效应、作用机制和暴露生物标志物研究中,详见表3。

**表3:** MSI技术在环境污染物毒性效应研究中的应用

Pollutants	Organism	Range of *m/z*		Detected mode	Ref.
Fipronil	zebrafish	650-	950	P and N	[56]
Graphene nanoparticles	*Eisenia fetida*	100-	1000	P	[57]
Chlorinated paraffins and hexabromocyclododecane	zebrafish	80-	1200	P and N	[6]
Bisphenol S	mouse liver	200-	1100	N	[58]
	mouse kidney				[59]
	mouse spleen				[60]
PM2.5	mouse placenta and fetuses	200-	1100	N	[61]
benzo[a]pyrene	mouse liver	140-	1100	P	[62]
Cd	mouse liver	120-	1800	P and N	[63]
Climbazole	zebrafish	100-	1000	P	[64]

P: positive ion mode; N: negative ion mode.

MSI技术用于研究环境污染物全身效应时主要针对体型较小的斑马鱼、蜜蜂和蚯蚓等模式生物。Liu等^[56]^采用MALDI-MSI技术发现氟虫腈暴露后斑马鱼体内PC、PS和PI含量明显下降,眼睛部位最为明显,显著变化脂质包括PC (34∶2)、PC (34∶1)、PC (34∶2)、PC (36∶4)、PC (38∶6)、PS (18∶0/22∶6)、PI (18∶0/20∶4)和PI (18∶1/20∶4)。Zhang等^[57]^采用此技术发现石墨烯纳米颗粒暴露会影响蚯蚓尾部区域丙氨酸和苯丙氨酸减少,头部区域脯氨酸、组氨酸和精氨酸增加,且不同部位谷氨酸变化与暴露剂量有关。Huang等^[6]^采用AFAI-DESI-MSI技术发现短链和中链氯化石蜡暴露会导致斑马鱼体内大多数内源性代谢物浓度降低,前者主要引发肝脏中PC和PE含量下降和甘油三酯(TG)累积,后者主要引发肝脏、肠道、心脏、大脑中多胺和肌苷相关代谢物显著降低;六溴环十二烷暴露可使得斑马鱼肌肉中脯氨酸、肾脏中肌酸和肌酐以及卵巢中甘油磷脂(PC和PE)含量升高。

基于空间代谢组学的环境污染物暴露对模式生物的器官毒性效应研究主要采用MALDI-MSI技术。蔡宗苇教授团队采用此技术对双酚S的靶器官毒性效应进行了全面解析,发现暴露后BALB/c裸鼠肝脏中PE、溶血PC(LPC)、溶血PE和溶血PS显著升高,PC和PS显著下调,且部分脂质呈非均匀变化^[58]^;肾皮质、髓质和肾盂中炎症相关神经酰胺和鞘磷脂(SM)上调,肾皮质中与结构脂质甘油磷脂、鞘脂和甘油酯变化趋势多样^[59]^;脾脏白质中炎症相关脂质也发生了显著变化^[60]^。该团队还发现PM2.5暴露后孕鼠胎盘和胎鼠脑组织中部分脂质含量上调且空间分布变化,并以性别特异性差异方式诱导子代认知和情绪障碍^[61]^。我们团队采用此技术表征了苯并[*a*]芘暴露小鼠不同靶器官脂质的变化特征,发现小鼠肝脏组织中16个PC、LPC和SM共三类结构脂质空间分布发生变化,且含量均下调^[62]^;脑组织皮层、海马、白质和丘脑4个脑区中甘油磷脂、甘油酯和脂肪酸含量也发生了变化,不同脑区差异脂质类别明显不同。此外,研究者们还发现Cd暴露会导致ICR小鼠肝脏中部分甘油二酯和甘油三酯含量下调^[63]^,氯咪巴唑暴露可显著影响斑马鱼卵巢中谷胱甘肽和脂肪酸代谢通路^[64]^。

总体来看,与代谢组学技术相比,基于MSI技术的空间代谢组学可原位表征生物体对污染物暴露的代谢应答情况,尤其是在小体型模式生物和异质性的组织和器官中。MSI技术为外源性污染物毒性效应研究开辟了新视角,通过与常规毒理学技术或其他组学技术相结合可对污染物毒性进行全面解读。

## 4 结论与展望

MSI技术在环境污染物分析及毒性效应研究中发挥着重要作用,主要用来提供外源性污染物和内源性代谢物在模式生物全身和特定组织中的含量和空间分布信息。不同离子化技术的工作原理、适用分析对象和成像空间分辨率等不同。采用MSI技术原位解析模式生物体内污染物的毒代动力学与代谢应答的毒性效应研究,有助于阐明污染物的毒性效应与作用机制,发现暴露生物标志物。尽管MSI技术表现出了独特的原位可视化分析的优势,但对于低含量、难电离的有机污染物和内源性代谢物的分析效果不佳。而且MSI技术本身也存在可重复性较差、数据采集时间长、化合物注释和鉴定难等问题。为此,迫切需要研发能够兼顾高分辨率和成像速度的新型高灵敏度的成像质谱,开发先进的质谱成像软件和海量数据的挖掘系统。此外,发展多模态成像技术,将MSI与其他分子成像技术和空间组学技术相结合,以最大限度地获取化学和生物信息,全面解析污染物的毒性。随着MSI成像技术的不断发展和完善,必将进一步推动环境污染物分析及毒理学研究,极大地拓展其应用领域。
